# Gut-Kidney Axis: Unraveling the Role of the Microbiome in Chronic Kidney Disease

**DOI:** 10.3390/biomedicines14010109

**Published:** 2026-01-06

**Authors:** Mihai Rusu, Cristian Ichim, Paula Anderco, Andreea Pălăștea, Adrian Boicean

**Affiliations:** 1 Faculty of Medicine, Lucian Blaga University of Sibiu, 550169 Sibiu, Romania; dr.mihairusu@yahoo.com (M.R.); cristian.ichim@ulbsibiu.ro (C.I.); adrian.boicean@ulbsibiu.ro (A.B.); 2 Predeal Sanatorium for Neurological and Psychiatric Conditions, 505300 Predeal, Romania

**Keywords:** gut–kidney axis, chronic kidney disease, gut microbiota, uremic toxins

## Abstract

Chronic kidney disease (CKD), which affects over 850 million individuals globally, is increasingly regarded as a systemic condition in which the gut microbiota represents a key pathogenic node. This review provides an integrated overview of mechanistic, translational and clinical data implicating the gut–kidney axis in CKD. The CKD-associated microbiota displays a characteristic dysbiosis, marked by depletion of short-chain fatty acid–producing commensals, overgrowth of proteolytic and urease-expressing taxa and disruption of epithelial barrier integrity. These disturbances favor the generation and systemic accumulation of gut-derived uremic toxins, most notably indoxyl sulfate, p-cresyl sulfate, indole-3-acetic acid and trimethylamine-N-oxide, which promote endothelial dysfunction, vascular calcification, fibrosis and chronic inflammation, thereby hastening renal function loss and heightening cardiovascular risk. Microbiome-directed interventions, including dietary modification, prebiotics, probiotics, synbiotics, intestinal dialysis, fecal microbiota transplantation, gut-acting sorbents and nephroprotective phytochemicals, are summarized with emphasis on their effects on uremic toxin burden and clinical surrogates. System-level implications of the gut–kidney axis for cardiovascular disease, immunosenescence and sarcopenia are discussed, together with future priorities for integrating multi-omics profiling and precision microbiome-based strategies into nephrology practice.

## 1. Introduction

Chronic kidney disease (CKD) affects close to 850 million people globally and ranks as one of the major causes of morbidity and mortality on a worldwide scale [1]. Traditional pathophysiological models emphasize hemodynamic overload, metabolic derangements and progressive fibrosis as the main drivers of renal functional decline [2,3]. In this framework, the gut microbiome, comprising bacteria, fungi and archaea has long been regarded as a secondary player in CKD onset and progression, rather than a central determinant [4,5].

Renal function is increasingly recognized as being modulated by intestinal barrier integrity, immune mediators and microbially derived metabolites. These signals communicate along a bidirectional pathway termed the gut–kidney axis [6,7]. Dysbiosis, defined as a disruption in gut microbial ecology, has been consistently associated with elevated uremic toxin levels, cardiovascular complications and systemic inflammation [8]. This conceptual shift moves the kidney from an isolated-organ model to a component of a complex metabolic and immunologic network. It is largely driven by advances in metagenomics, metabolomics and systems biology that have illuminated the gut–kidney axis [9,10,11,12].

By altering the normal host–microbe equilibrium, CKD leads to gut dysbiosis, which in turn feeds back negatively and promotes the advancement of the disease [13]. Elevated levels of uremic toxins, mechanical alterations of the gastrointestinal tract and changes in colonic transit are frequently observed in CKD [14,15]. In parallel, reduced renal excretion leads to accumulation of urea in the gut, which enhances urease activity, increases luminal pH and promotes systemic inflammation [16,17,18]. Over time, these changes drive compositional shifts in the gut microbiota, abnormal metabolite production and increased intestinal permeability [16,17,18]. Histological analyses of intestinal tissue from CKD patients have shown reduced villous height, crypt elongation and inflammatory cell infiltration of the lamina propria [19]. Collectively, these observations support the existence of a complex, bidirectional gut–kidney axis in which renal impairment and intestinal dysbiosis perpetuate one another.

Beyond the kidney, intestinal dysbiosis has also been linked to liver injury, altered bile acid profiles and metabolic reprogramming in experimental CKD models [20]. These changes may further amplify renal damage through a broader gut–liver–kidney axis. Overall, accumulation of uremic toxins secondary to renal impairment perturbs gut microbial composition and metabolism and in turn, dysbiosis-driven production of additional uremic solutes exacerbates kidney injury, establishing a self-perpetuating vicious cycle [21].

Metabolic dysfunction is increasingly framed as a systemic, endocrine–immune disorder with direct renal consequences, captured by the emerging concept of metabolic dysfunction–associated kidney disease [22,23]. In this framework, hormonal and adipose tissue–derived signals, particularly hyperinsulinemia/insulin resistance and adipokine imbalance, contribute to glomerular hyperfiltration, microvascular injury, inflammatory activation and fibrotic remodeling, thereby promoting CKD onset and progression in individuals with obesity- and MetS-related phenotypes [22,23]. In parallel, dysregulated endocrine axes, including mineralocorticoid/RAAS activation and sex hormone perturbations, further modulate vascular and renal injury pathways, reinforcing the cardio–renal consequences of metabolic dysfunction [24,25].

Recent multi-omics studies have reinforced the concept that the intestinal ecosystem is a major determinant of CKD progression [9,10,11,12]. An expanding body of research shows that shifts in the gut microbiota’s structure and metabolic activity can strongly shape human health and illness [26,27,28]. It is becoming increasingly evident that disturbances in microbial balance may exert direct effects on renal function and disease processes. Impaired kidney function has been increasingly linked to the accumulation of gut-derived uremic solutes, establishing the gut microbiome as a relevant contributor to renal and cardiovascular pathology [14,29,30].

Moreover, CKD is associated with oxidative stress, endotoxemia, chronic low-grade inflammation and a high burden of cardiovascular comorbidities, processes increasingly linked to disturbances of the gut ecosystem [29,30]. Given this framework, the aim of the present review is to integrate and examine existing research on the relationship between the human gut microbiome and renal pathology. Particular emphasis is placed on how shifts in gut microbial composition and function influence CKD development, progression and complications. In addition, the review critically evaluates whether gut dysbiosis acts primarily as a causal driver, an amplifier or a consequence of CKD, and highlights emerging microbiome-targeted strategies with potential nephroprotective implications.

## 2. Alterations of the Gut Microbiota in CKD

When gut microbial communities lose their structural and functional balance, disruption of the commensal ecosystem leads to intestinal dysbiosis with impaired barrier integrity and increased permeability [31,32,33]. In this context, viable bacteria and their products can translocate from the intestinal lumen to extraintestinal sites, including the kidney, a process favored by dysbiosis, bacterial overgrowth and reduced host immune defenses [34,35,36]. In CKD, dysbiosis arises from both the uremic milieu and disease-related lifestyle and pharmacologic factors, producing characteristic taxonomic and functional alterations [6,15,37].

The gut microbiota is a major source of uremic solutes such as indoxyl sulfate (IS), p-cresyl sulfate (p-CS) and trimethylamine-N-oxide (TMAO) in CKD [14,29,30]. Rising systemic urea levels, in turn, feedback on the intestinal environment and further alter microbial composition, establishing a bidirectional, maladaptive loop between uremia and dysbiosis [38]. Uremic solutes are implicated in numerous CKD-related problems, including anemia of kidney disease, pruritus, persistent tiredness, mineral-bone abnormalities, neurological deficits and cardiovascular disorders [37].

Taxonomically, CKD is often associated with reduced microbial α-diversity and depletion of beneficial saccharolytic genera such as *Lactobacillus*, *Prevotella* and *Bifidobacterium* [39,40,41]. In parallel, proteolytic, urease-producing or pathobiont-enriched taxa, including *Enterobacteriaceae*, *Clostridium* spp., *Desulfovibrio* and *Enterococcus*, become overrepresented [42,43,44,45]. This shift toward protein-fermenting and inflammation-associated microbes reflects the combined effects of increased luminal urea, altered pH, reduced dietary fiber intake, constipation, exposure to antibiotics and phosphate or potassium binders and slowed intestinal transit, all of which are common in CKD [46,47].

Functionally, the CKD microbiome exhibits enhanced proteolytic fermentation and diminished short-chain fatty acid (SCFA) production [48,49,50]. As renal function declines, key SCFA-producing bacteria like *Faecalibacterium prausnitzii* and *Roseburia intestinalis* diminish substantially, leading to lower butyrate levels and weakening epithelial metabolism, mucosal defense and barrier stability [51,52,53]. Metatranscriptomic studies further show increased expression of genes involved in aromatic amino acid metabolism and sulfur compound production, pathways that generate precursors of IS, p-CS and other gut-derived uremic solutes [9,54,55].

With progressive loss of renal function, rising uremic burden alters the intestinal environment and is associated with features of impaired barrier integrity [56]. This breakdown of barrier integrity allows microbial toxins and other injurious metabolites to enter the systemic bloodstream, thereby amplifying inflammation throughout the body and hastening kidney damage [56].

Together, these compositional and functional alterations define a CKD-specific dysbiotic signature characterized by: reduced microbial richness, expansion of urease- and protease-producing taxa, depletion of SCFA-producing commensals and enhanced microbial capacity for uremic toxin generation [9,38,39]. This constellation not only mirrors declining renal function but also contributes to its progression through sustained metabolic, inflammatory and immune activation.

## 3. Pathophysiological Mechanisms of the Gut–Kidney Axis

The diseased cross-talk between gut microbiota and renal tissue, commonly termed the gut–kidney axis, is increasingly implicated in several kidney-related conditions, such as CKD, acute kidney injury, high blood pressure, nephrolithiasis, IgA nephropathy and in patients receiving hemodialysis or peritoneal dialysis [57,58]. Advances in metagenomics and metabolomics have substantially improved our ability to characterize the microbiome and its metabolites in these settings, highlighting the contribution of the gut–kidney axis across different kidney pathologies [9,20,38,58]. Nonetheless, the mechanisms through which the gut microbial community interacts with the host remain only partly understood and advancing our knowledge of this interplay will likely elucidate the underlying causes and pathways of disease [28,59,60].

### 3.1. Intestinal Barrier Dysfunction and Endotoxemia

Patients with CKD commonly display a “leaky gut” pattern marked by heightened intestinal permeability, a condition worsened by uremia, edema of the intestinal wall and ischemic changes affecting the gut mucosa [61]. On a molecular scale, CKD is accompanied by diminished and disordered tight-junction components like ZO-1, claudins and occludin, causing the paracellular barrier to loosen and become more permeable [62,63]. As a result, endotoxins such as lipopolysaccharide (LPS), along with bacterial products and, occasionally, viable microorganisms, can cross the mucosal barrier and enter the systemic circulation [34,35,61,62,63].

Uremia itself aggravates these barrier defects. Rising blood urea levels in CKD enhance diffusion of urea into the intestinal lumen, where it is hydrolysed by bacterial urease to ammonia and ammonium hydroxide [64,65]. These compounds increase luminal pH, damage epithelial cells and further disrupt tight junction architecture, thereby amplifying paracellular permeability [16,17,18]. Elevated circulating levels of LPS correlate strongly with higher serum concentrations of TNF-α and IL-6, supporting the presence of endotoxemia and amplifying the inflammatory burden in CKD [62,63,66].

### 3.2. Immune Activation and Chronic Inflammation

Increased intestinal permeability allows bacterial constituents and LPS to cross into the systemic circulation, where they provoke dysregulated immune activation at both the mucosal surface and throughout the body [67,68]. LPS engages Toll-like receptor 4 on innate immune cells, subsequently triggering nuclear factor-kappa B (NF-κB) signaling and inducing the expression of pro-inflammatory mediators such as IL-6, TNF-α and various chemokines [62,63,66]. In parallel, pathobionts stimulate dendritic cells, which polarize naive T cells toward Th17 and Th1 phenotypes, enhancing production of IL-17, IFN-γ and other cytokines that perpetuate low-grade systemic inflammation [69,70].

Chronic endotoxemia thereby drives renal inflammation, macrophage activation and cytokine release within the kidney interstitium [14,71]. Activation of NF-κB and JAK–STAT signaling pathways in tubular epithelial and endothelial cells contributes to apoptosis, extracellular matrix deposition and interstitial fibrosis, providing a mechanistic bridge between gut-derived inflammatory stimuli and structural kidney damage [72,73,74,75,76]. This leaky gut state establishes a vicious cycle in which inflammation further destabilizes the gut microbiome, aggravating dysbiosis and contributing to the progression of kidney disease [61,77,78].

### 3.3. Gut-Derived Uremic Toxins and Vascular–Renal Injury

**Figure 1:**
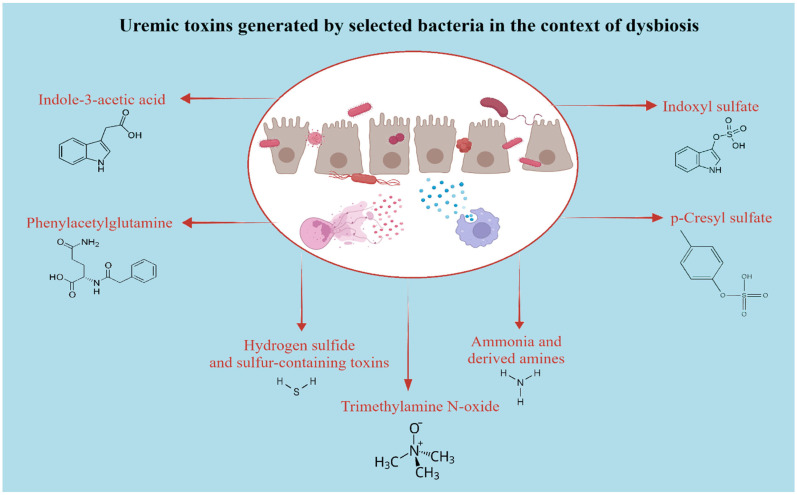
Uremic toxins generated by selected bacteria in the context of dysbiosis. (Major gut-derived uremic toxins produced under dysbiotic conditions in CKD. Shown metabolites arise from microbial metabolism of dietary substrates and contribute to vascular and renal injury.)

Beyond barrier and immune perturbations, dysbiosis in CKD is characterized by a metabolic shift from saccharolytic to proteolytic fermentation, favoring the generation of nitrogenous and phenolic compounds [44,79,80]. As summarized in Figure 1, these toxins include protein-bound metabolites and sulfur-containing compounds generated by dysbiotic microbial metabolism. Key protein-bound uremic solutes are all generated during microbial degradation of proteins in the colon [29,81]. Intestinal microorganisms transform ingested tryptophan into indole, which is then further metabolized to IAA locally in the gut and to IS after hepatic conversion [82]. Concomitantly, p-CS results from bacterial catabolism of the aromatic amino acids tyrosine and phenylalanine. These toxins accumulate as kidney function declines and are poorly cleared by conventional dialysis because of their strong albumin binding [29,45,82,83].

**Figure 2:**
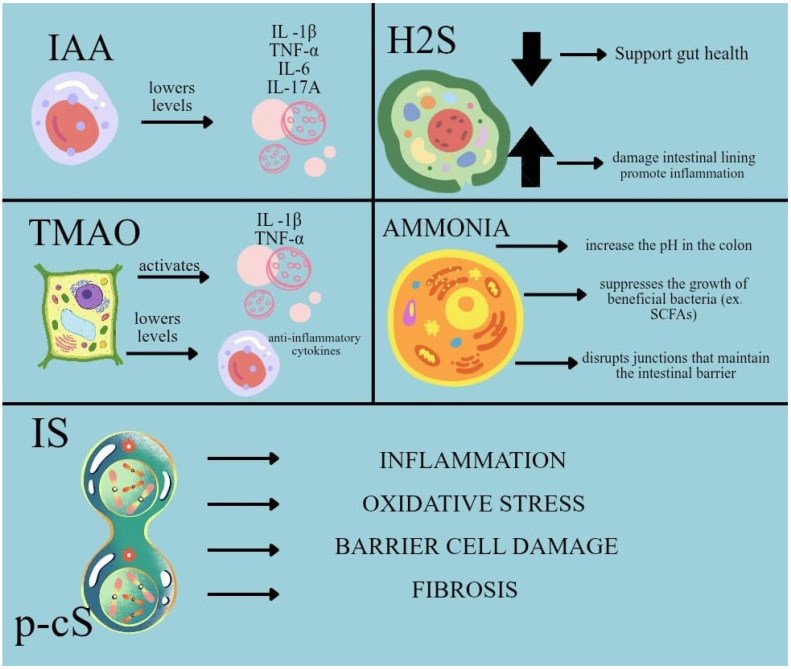
Mechanistic effects of gut-derived uremic solutes on intestinal barrier integrity and vascular–renal injury. (Schematic overview of key downstream pathways linking dysbiosis-associated microbial metabolites to CKD progression, highlighting effects on epithelial barrier disruption, inflammatory cytokine signaling, oxidative stress and pro-fibrotic responses that collectively contribute to vascular and renal injury. Abbreviations: IAA—indole-3-acetic acid, IS—indoxyl sulfate, p-CS—p-cresyl sulfate, TMAO—trimethylamine N-oxide, H_2_S—hydrogen sulfide.)

As outlined in Figure 2, gut-derived uremic solutes promote intestinal barrier injury, inflammatory activation, oxidative stress and pro-fibrotic remodeling, linking dysbiosis to vascular and renal damage.

Both IS and IAA, which originate from tryptophan metabolism, act as ligands for the aryl hydrocarbon receptor and activation of this pathway has been linked to atherogenesis, vascular inflammatory responses and heightened oxidative stress [29,82,83]. Engagement of aryl hydrocarbon receptor by IS and IAA promotes the expression of pro-inflammatory genes and oxidative enzymes, augments reactive oxygen species (ROS) production and enhances tissue factor expression, thereby fostering a pro-thrombotic and pro-atherogenic milieu [84,85,86]. Evidence from cell and animal models indicates that IS stimulates leukocyte–endothelial interactions by upregulating E-selectin, ICAM-1 and VCAM-1, while its transport into cells via organic anion carriers further intensifies ROS formation within endothelial and tubular compartments [29,87,88,89].

IS and p-CS also directly promote vascular calcification and vascular smooth muscle cell (VSMC) proliferation [90,91]. Experimental data indicate that these solutes stimulate osteogenic transdifferentiation of VSMCs, upregulate bone-related proteins and increase calcium deposition within the vascular wall [45,90,92,93]. These changes contribute to arterial stiffness and medial calcification, hallmarks of the accelerated vascular aging observed in CKD.

TMAO, generated from dietary choline, phosphatidylcholine and L-carnitine by gut bacteria and hepatic flavin monooxygenases, constitutes another important link between dysbiosis, kidney disease and cardiovascular events [56,94,95]. TMAO has been shown to increase the expression of adhesion molecules, promote endothelial nitric oxide synthase uncoupling and enhance foam cell formation, thereby driving endothelial dysfunction and atherosclerosis [96,97,98]. Elevated TMAO levels are strongly associated with incident CKD, faster renal function decline and higher mortality risk, even after adjustment for traditional risk factors [94,95].

### 3.4. An Integrated Gut–Vascular–Kidney Axis

Taken together, barrier disruption, immune activation and toxin accumulation form a pathophysiological triad that links the intestinal lumen to renal and vascular compartments [99,100]. Increased intestinal permeability permits bacterial products and toxins to reach the systemic circulation, where innate and adaptive immune responses maintain low-grade inflammation, while protein-bound solutes such as IS, p-CS, IAA and TMAO directly damage endothelial and tubular cells, foster fibrosis and accelerate vascular calcification [12,47,78,101]. These interconnected processes underpin a broader “gut–vascular–kidney” axis in which dysbiosis simultaneously accelerates CKD progression and amplifies cardiovascular risk.

At the vascular level, gut-derived uremic toxins and inflammation-driven immune activation exert direct and indirect effects on endothelial and smooth muscle cell function, thereby linking intestinal dysbiosis to systemic vascular pathology [102]. Protein-bound solutes impair endothelial nitric oxide bioavailability, promote oxidative stress and disrupt endothelial barrier function, resulting in endothelial dysfunction and increased arterial stiffness [103,104]. In parallel, chronic endotoxemia and low-grade inflammation enhance leukocyte adhesion, activate vascular inflammatory signaling pathways and accelerate atherogenesis, further amplifying cardiovascular risk in CKD [105,106]. Moreover, uremic toxins stimulate osteogenic transdifferentiation of vascular smooth muscle cells, contributing to medial calcification and structural vascular remodeling, processes that are tightly associated with arterial stiffening and adverse cardio–renal outcomes in advanced CKD [107,108].

Early CKD may be accompanied by milder barrier defects and modest elevations in gut-derived solutes, whereas advanced stages and dialysis are characterized by more pronounced endotoxemia, higher circulating levels of IS, p-CS and TMAO and deeper perturbations of immune and vascular homeostasis [109,110,111,112]. Understanding when and how these pathways become operative is crucial for identifying therapeutic windows in which microbiota-targeted interventions, dietary modulation or pharmacologic strategies aimed at gut-derived toxins could most effectively modify disease trajectory.

## 4. Microbiome-Targeted Therapeutic Strategies in CKD

Intestinally derived metabolites display a dual impact on renal function. While SCFAs sustain epithelial cohesion and reinforce gut barrier competence, nitrogenous and phenolic molecules generated during protein fermentation, including ammonia and phenols produced by urease-positive microbes, foster systemic inflammatory responses and facilitate kidney injury [113,114]. In CKD, impaired renal clearance amplifies the accumulation of these toxic solutes, reinforcing a self-perpetuating cycle of metabolic and tissue damage [114]. Bacteria with proteolytic activity, such as *Bacteroides* and *Clostridium*, become overrepresented in CKD and are responsible for producing ammonia, various amines, thiols, phenols and indole derivatives [12]. Urea derived from protein catabolism is reconverted to ammonia in the colon via bacterial urease and rising blood urea levels in CKD enhance its diffusion into the gut and increase ammonia generation [37,115]. Excess ammonia raises luminal pH, injures epithelial cells and disrupts tight junction proteins, thereby promoting a “leaky gut” and facilitating translocation of microbial products that fuel IL-6– and TNF-α–mediated inflammation [37,116].

### 4.1. Dietary Modulation of the Gut Microbiota

Dietary patterns characterized by a higher proportion of plant-based foods and a lower contribution of animal protein appear to exert a more integrative influence on gut microbial ecology and uremic toxin generation than individual nutrients considered in isolation [117,118,119,120]. Plant-dominant dietary patterns, including the Mediterranean and DASH diets, have been associated with enhanced microbial diversity, increased short-chain fatty acid production and a lower generation of precursors of protein-bound uremic toxins such as IS and p-CS [117,118,119,120].

In contrast, protein-rich dietary patterns in the uremic milieu promote proteolytic fermentation and the expansion of toxin-generating bacteria, thereby increasing the production of metabolites such as IS, p-CS and ammonia and potentially amplifying systemic toxicity and cardiovascular risk in CKD [121,122]. The balance between dietary protein intake and fermentable fiber availability may therefore represent a key determinant of microbial metabolic output and may partly explain heterogeneous findings in fiber-focused interventions, supporting the clinical relevance of dietary pattern–based approaches rather than isolated dietary components [117,118,121,122].

### 4.2. Probiotics, Prebiotics and Synbiotics

In this context, prebiotic compounds such as inulin and resistant starch have been shown to selectively expand SCFA-producing taxa, while specific probiotic strains of Bifidobacterium and Lactobacillus may reduce the abundance of toxin-generating bacteria [123,124,125]. However, these effects are strain-specific and primarily supported by changes in microbiome composition and circulating metabolites rather than by clinical outcome data. At the same time large-scale randomized controlled trials of probiotic or synbiotic interventions in CKD remain limited and available studies are characterized by short follow-up periods, modest sample sizes and heterogeneity in strain composition, with a predominant focus on surrogate biochemical or inflammatory endpoints rather than hard renal or cardiovascular outcomes [126,127,128].

Consequently, current evidence is insufficient to justify routine probiotic use in CKD and future trials should incorporate rigorous dietary control and standardized selection and dosing of probiotic strains to allow meaningful comparisons across studies. At present, probiotic, prebiotic and synbiotic interventions remain adjunctive and investigational in CKD, with heterogeneous trial designs and limited evidence on hard renal or cardiovascular outcomes; therefore, they are not guideline-endorsed as disease-modifying therapy [129].

Restoring a balanced intestinal microbiota is central to alleviating gut dysbiosis and its downstream effects on immune dysfunction, inflammation and kidney injury. Diet is a major determinant of microbial composition [130,131,132]. In advanced CKD, restrictions aimed at preventing hyperkalemia and oxalate overload, together with chronic use of phosphate binders, antibiotics and high-salt intake, can profoundly disturb microbiota structure and function, including reductions in Lactobacillus and altered Th17 responses [17,133,134].

Accordingly, several microbiota-targeted strategies, including probiotics, prebiotics and synbiotics, have been evaluated as adjunctive interventions in CKD [135]. Probiotics have been reported to lower circulating uremic solutes and partially restore microbial balance in selected CKD populations [135]. However, their effects are highly strain-specific and largely supported by experimental data and small clinical studies, with inconsistent results in advanced CKD and dialysis settings, where interpretation is further complicated by comorbidity burden and dialysis-related confounders [136,137,138,139,140,141]. Although serious adverse events are uncommon, careful patient selection and monitoring are warranted when microbiota-based interventions are considered in advanced CKD.

Prebiotics, non-digestible fiberrs that selectively stimulate beneficial taxa, promote SCFAs production, strengthen barrier function, modulate inflammation, as well as improve glucose and lipid metabolism [140]. In both adult CKD and pediatric end-stage-renal-disease, prebiotic supplementation and increased dietary fibre have been associated with reductions in serum urea nitrogen and circulating uremic toxins [141,142]. Synbiotics, combining probiotics and prebiotics, appear to offer additive benefits, including lower urinary toxin levels and favorable shifts towards increased *Bifidobacterium* and reduced *Ruminococcaceae*, with only exploratory signals suggesting a possible slowing of CKD progression [70]. Complementary approaches such as SCFA supplementation show experimental renoprotective effects by attenuating renal inflammation and apoptosis [143]. Accordingly, current microbiota-based interventions should be viewed as adjunctive investigational strategies rather than disease-modifying therapies in CKD.

### 4.3. Intestinal Dialysis and Nitrogen-Binding Strategies

The colon is a key site for nitrogen waste disposal and has been suggested as a potential therapeutic pathway in CKD [144]. The idea of clearing metabolic by-products outside the kidneys via the intestinal mucosa has been recognized for many years and modern approaches include oral nitrogen-binding agents such as oxidized starch to reduce gastrointestinal nitrogen load [145]. Experimental work in the 1970s introduced the idea of “colon dialysis” as a simplified alternative to hemodialysis and peritoneal dialysis and this technique has since been applied clinically, including in a pediatric uremic case [146].

More recent clinical studies support the therapeutic potential of this strategy [147]. One single-center trial in 88 patients reported that colonic dialysis achieved biochemical control and symptom relief broadly comparable to hemodialysis and peritoneal dialysis in CKD stages 4–5, while another study suggested that colonic dialysis may help preserve kidney function in CKD stages 3–5, potentially by preventing intestinal dysbiosis [144,148]. These observations reinforce the importance of gut-derived toxins in CKD progression, in line with emerging data on the contribution of intestinal metabolites to renal injury [149].

### 4.4. Fecal Microbiota Transplantation and Gut-Acting Adsorbents

More intensive interventions include fecal microbiota transplantation (FMT), currently established mainly for recurrent *Clostridioides* difficile infection, but still limited by ethical, safety and logistical concerns and with sparse data in renal patients [150,151,152]. Oral carbon adsorbent therapy with AST-120, which binds indole and IS in the gut, has been reported to reduce uremic toxin levels, delay dialysis initiation and mitigate tubular injury, although most evidence comes from Japanese cohorts [153,154,155]. Finally, kidney transplantation itself induces major shifts in the gut microbiota, adding further complexity to the post-transplant gut–kidney axis [152,156].

In animal models of CKD, FMT has been shown to restore microbial diversity, attenuate renal inflammation and lower circulating levels of IS and p-CS [157,158]. Although its clinical use in CKD remains largely experimental, FMT represents an important frontier in microbiome-targeted nephrotherapy.

Recent experimental studies indicate that microbiota-targeted interventions, including FMT, can remodel the uremic metabolite profile and are associated with reduced renal fibrosis, with decreased expression of profibrotic markers in rodent models of kidney injury and CKD [159,160,161,162]. Nevertheless, unresolved concerns regarding long-term safety, immune compatibility and the risk of pathogen transmission currently preclude FMT from being adopted as a routine therapeutic strategy in clinical nephrology.

### 4.5. Phytochemicals and Plant-Based Nephroprotection

Medicinal plants have been employed for many generations and a substantial proportion of contemporary pharmaceuticals are derived from natural substances that were first used in traditional healing systems [163]. Their global use is steadily rising, mainly because herbal products are relatively inexpensive and usually well tolerated. Consequently, phytochemicals and plant-based remedies are becoming increasingly important in medical practice as naturally sourced agents that can be incorporated as dietary supplements, herbal therapies or components of a balanced diet [164,165]. These preparations include a wide variety of organic molecules, such as phenols, saponins, glycosides, flavonoids, alkaloids, tannins, steroids and terpenoids, that exert specific biological effects and shape the gut microbiota by supporting beneficial microbial populations while restraining potentially harmful species [7]. These molecules are unevenly distributed across plant tissues and cellular compartments and display a wide range of biological activities, including antioxidant, chemopreventive, neuroprotective, cardioprotective and immunomodulatory effects [166].

From a renal perspective, phytochemicals are particularly attractive because of their nephroprotective potential. By attenuating oxidative stress, a key driver of glomerular and tubular injury and an important contributor to hypertension, inflammation and endothelial dysfunction in CKD, they may help slow disease progression. Phenolic compounds, which represent the most extensive category of plant-derived metabolites, are considered some of the most potent antioxidant molecules [167]. This class includes phenolic acids, tannins, stilbenes, lignans and flavonoids, the latter being produced in response to microbial challenge and exhibiting antibacterial, diuretic, natriuretic and nephroprotective effects in both acute kidney injury and CKD [168].

Flavonoids are widely distributed in culinary plants, being particularly plentiful in herbs, cereals and citrus fruits, among other dietary sources [169]. Alkaloids, nitrogen-containing secondary metabolites, are extensively employed as pharmacologically active components in numerous drugs, whereas tannins have been linked to blood pressure–lowering properties and antimicrobial activity [170]. Anthocyanins, a subclass of flavonoids responsible for the characteristic coloration of many fruits, leaves and flowers, are abundant in berries and cherries and can modulate gut microbial communities, as most of these compounds bypass absorption in the upper gastrointestinal tract and reach the colon, where they are further transformed by resident bacteria [171].

The structured discovery and isolation of phytochemicals with nephroprotective potential depend on systematic screening approaches and bioactivity-guided experimental studies. Globally, numerous plants and plant-derived compounds are being investigated as candidate therapies or adjuncts for the management of kidney diseases.

## 5. From Mechanisms to Practice: Clinical Evidence

A growing body of clinical and translational research has started to test whether the mechanistic insights into the gut–kidney axis can be translated into meaningful renal and cardiovascular benefits in patients with CKD [47,172,173]. Several small-scale trials suggest that microbiota-targeted interventions can modestly improve surrogate renal outcomes, including stabilization of eGFR and reductions in C-reactive protein, serum IS and p-CS [128,172,174]. More recent randomized controlled trials have reinforced these preliminary observations, lending additional support to the clinical relevance of microbiome modulation in CKD [126,175,176].

There is growing interest in metabolomic fingerprinting as a quantitative readout of therapeutic efficacy, reductions in IS, p-CS and TMAO being frequently paralleled by shifts in other gut-derived metabolites such as hippurate and phenylacetylglutamine, indicating broad metabolic reprogramming in response to microbiota-focused interventions [177,178,179,180]. Dietary patterns and microbiota-focused interventions that modulate TMAO concentrations have been linked to improvements in surrogate measures of vascular function, including indices of arterial stiffness and endothelial function, underscoring the cardiovascular importance of the gut microbiome [181,182,183].

Furthermore, dysbiotic gut microbiota are believed to contribute to the cardio–renal syndrome through sustained generation of uremic toxins [184]. Among these, the gut-derived IS and p-CS have been extensively studied over the past decade, with robust experimental and clinical evidence supporting their nephrovascular toxicity [185]. Both IS and p-CS are generated as end-products of microbial breakdown of dietary proteins within the colon.

Renoprotective agents such as SGLT2 inhibitors and GLP-1 receptor agonists may interact with gut-directed therapies, as emerging data suggest that these drugs can modulate gut microbial composition, most consistently by increasing SCFAs producing bacteria for SGLT2 inhibitors, with more heterogeneous but broadly favorable microbiota changes reported for GLP-1 receptor agonists [186,187,188]. This pharmacologic–microbiome convergence supports the view that modulation of the gut ecosystem could augment the benefits of standard CKD therapies and potentially reduce residual cardio–renal risk.

Emerging data also indicate that specific gut-derived metabolites can serve as non-invasive biomarkers for CKD staging and prognosis and that integrated metabolomic and metagenomic approaches enable increasingly personalized risk stratification. Several metabolomic studies have linked higher circulating or urinary levels of microbial co-metabolites, such as p-cresol conjugates, indole-3-acetic acid/indoleacetate and phenyl sulfate, with faster CKD progression, albuminuria progression or adverse renal outcomes, although urinary metabolomic signatures of rapid eGFR decline remain largely exploratory [189,190,191].

The impact of the oral activated carbon adsorbent AST-120 on the gut milieu has been explored primarily in experimental models of renal failure [192]. In rat studies, AST-120 partially restored Lactobacillus abundance, improved tight junction protein expression and mucin-secreting goblet cell proportions, while reducing circulating indoxyl sulfate and pro-inflammatory cytokines, suggesting a beneficial effect on intestinal barrier function mediated by intraluminal toxin binding [129,192]. However, translation into clinical benefit has been inconsistent. Two large placebo-controlled randomized trials in CKD failed to demonstrate significant effects on composite renal endpoints or eGFR decline, likely reflecting limitations related to event rates, follow-up duration, regional practice patterns and adherence [193,194,195,196,197].

Other gut-acting agents have shown similar, predominantly biochemical effects. The phosphate binder sevelamer has been reported to lower circulating concentrations of the gut-derived uremic toxin p-cresol, suggesting indirect anti-inflammatory actions mediated through modulation of the intestinal milieu [198]. While these findings support the concept that intestinal sorbents and binders can influence uremic toxin burden, consistent benefits on hard renal or cardiovascular outcomes have not been demonstrated.

Beyond these CKD-focused agents, several commonly used non-antibiotic drugs, including SGLT2 inhibitors, metformin, nonsteroidal anti-inflammatory drugs, antipsychotics, proton-pump inhibitors, laxatives and statins, have been associated with reproducible shifts in gut microbiota composition [199,200]. These drug-induced microbial changes are not uniformly detrimental and may, in some cases, enhance therapeutic efficacy. Ongoing work dissecting drug–microbiome–host interactions is expected to refine existing therapies, support the development of microbiome-informed drugs and clarify the contribution of the microbiota to drug–drug interactions [201]. The main studies linking the gut microbiome to CKD incidence, progression and response to microbiota-targeted interventions are summarized in Table 1.

## 6. Conclusions

The gut–kidney axis has emerged as a central mediator of CKD, reshaping our understanding of renal pathophysiology from a primarily organ-centric model to an integrated, multisystem network. Mounting evidence from multi-omics studies, longitudinal cohorts and interventional trials demonstrates that intestinal dysbiosis is not merely a consequence of declining kidney function but an active contributor to uremic toxin generation, immune dysregulation, endothelial dysfunction and cardiovascular morbidity.

Across CKD stages, patients exhibit a characteristic dysbiotic signature marked by depletion of SCFA-producing commensals, expansion of proteolytic and urease-producing taxa, impaired barrier integrity and enhanced microbial capacity for producing indoxyl sulfate, p-cresyl sulfate and TMAO. These metabolic and structural perturbations propagate systemic inflammation, vascular injury and fibrosis, creating a self-perpetuating cycle that accelerates renal decline and amplifies extra-renal complications.

Clinical studies increasingly support the translational relevance of these mechanisms. Diet, synbiotics and specific microbiome-modulating interventions can reduce circulating uremic toxins, improve endothelial function and, in selected contexts, slow eGFR decline. However, most trials remain small, heterogenous and focused on surrogate biomarkers rather than hard renal or cardiovascular outcomes, underscoring the need for rigorous randomized controlled studies with standardized microbial, metabolomic and dietary assessments.

Emerging tools, including metagenomic risk profiling, microbial–host interaction mapping and integration of metabolomics with proteomic mediation, offer promising avenues for personalized prediction of CKD progression and therapeutic responsiveness. Drug–microbiome interactions further suggest that future nephrology therapies may combine pharmacologic agents with targeted microbial modulation to optimize efficacy and mitigate toxicity.

Looking forward, the most impactful advances will likely arise from integrative strategies that combine dietary optimization, microbiome-directed therapies and precision nephrology approaches rooted in multi-omics characterization. Such frameworks have the potential not only to modulate uremic toxin burden and inflammation but also to address the systemic manifestations of CKD, including cardiovascular dysfunction, immune aging and sarcopenia.

## Figures and Tables

**Table 1:** Major Human Studies Defining the Gut–Kidney Axis in CKD [Abbreviations: BUN—Blood urea nitrogen, CKD—Chronic kidney disease, eGFR—Estimated glomerular filtration rate, IS—Indoxyl sulfate, LC–MS—Liquid chromatography–mass spectrometry, p-CS—p-Cresyl sulfate, TMAO—Trimethylamine N-oxide].

Study Type/Population	Microbiome Exposure/Intervention	Kidney Outcomes	Synthesized Gut–Kidney Axis Finding (Conservative, Evidence-Based)	Source
OBSERVATIONAL COHORT STUDIES—MICROBIOME STRUCTURE & TOXINS				
Prospective cohort, 240 non-dialysis CKD stage 2–5 patients + controls	Shotgun metagenomics integrated with uremic toxins and dietary data	CKD severity, longitudinal eGFR	CKD is associated with characteristic dysbiotic features enriched in uremic toxin–producing pathways, with higher circulating indoxyl sulfate and p-cresyl sulfate levels associated with lower eGFR and greater disease severity.	[11]
Prospective cohort, 343 high–cardiovascular-risk adults	16S rRNA sequencing focusing on butyrate-producing taxa	CKD trajectory, microbial richness	In older adults at high CV risk, gut microbiota composition and alpha-diversity metrics differed between 1-year CKD trajectory groups, supporting an association between microbial community features and CKD progression/incidence over short follow-up.	[202]
Population-based cohort, 6556 adults	Shotgun metagenomics, α- and β-diversity	Incident CKD	Lower baseline microbial α-diversity is associated with increased long-term risk of incident CKD, suggesting microbiome diversity as a population-level marker of renal vulnerability.	[203]
METABOLITE-FOCUSED COHORTS—TMAO & UREMIC TOXINS				
Prospective cohort, 521 CKD patients	Plasma TMAO	Mortality, renal outcomes	Elevated circulating TMAO is independently associated with higher mortality and adverse renal outcomes, identifying gut-derived metabolites as prognostic markers of cardio–renal risk.	[95]
CAUSAL INFERENCE STUDIES—MENDELIAN RANDOMIZATION				
Two-sample MR (196 gut taxa)	Genetically predicted microbial taxa	CKD risk	Genetic analyses suggest that specific gut microbial taxa (e.g., *Desulfovibrionales*) are associated with increased CKD risk, supporting a potential causal contribution beyond reverse causation.	[204]
Two-sample MR, East Asian cohorts	Genetically predicted taxa and metabolic modules	CKD onset, BUN, eGFR	Specific microbial taxa show genetically informed associations with CKD risk and renal traits, partly mediated by host proteins, supporting microbiome–host interaction pathways in CKD.	[205]
DIETARY INTERVENTION STUDIES				
Randomized prospective crossover trial, CKD stage IIIB–IV	Mediterranean diet and very-low-protein diet	IS, p-CS, intestinal permeability	Dietary patterns rich in plant-based components and protein restriction are associated with reductions in circulating uremic toxins and improvements in gut permeability markers, indicating modifiable microbiome-related metabolic pathways.	[206]
MICROBIOTA-DIRECTED INTERVENTIONS AND PHENOTYPE-SPECIFIC MICROBIOME SIGNATURES				
Single-blind, non-randomized, placebo-controlled trial, CKD stage IV–V	Synbiotic supplementation	eGFR, IS, inflammatory markers	Synbiotic supplementation reduced IS, while effects on eGFR decline were modest and not robust after correction, suggesting metabolic effects without consistent clinical renal outcome benefit.	[172]
Cross-sectional multi-omics study of CKD subtypes	Long-read sequencing + LC–MS metabolomics	CKD presence and etiology	Distinct microbiome–metabolite profiles are observed across diabetic, hypertensive and non-comorbid CKD, highlighting etiologic heterogeneity of gut–kidney associations.	[207]

## Data Availability

No new data were created or analyzed in this study.
